# Response of water and salt accumulation in vadose zone to groundwater level changes in coastal saline soils using HYDRUS-1D model

**DOI:** 10.1371/journal.pone.0348208

**Published:** 2026-05-06

**Authors:** Feng Li, Shihong Yang, Yi Xu, Xiyun Jiao, Zewei Jiang

**Affiliations:** 1 College of Hydraulic Engineering, Zhejiang Tongji Vocational College of Science and Technology, Hangzhou, China; 2 College of Agricultural Science and Engineering, Hohai University, Nanjing, China; 3 Hydrology and Water Resources Department, Nanjing Hydraulic Research Institute, Nanjing, China; 4 College of Soil and Water Conservation, Hohai University, Nanjing, China; Ardakan University, IRAN, ISLAMIC REPUBLIC OF

## Abstract

**Purpose:**

Coastal saline soils are highly vulnerable to salinization, posing a major threat to sustainable agriculture. This study investigates how fluctuations in groundwater depth regulate water and salt dynamics in the vadose zone of a typical coastal saline area in Nanpi County, Hebei Province, China. The research focuses on quantifying changes in soil water storage and salt accumulation under different groundwater scenarios.

**Methods:**

Field monitoring was combined with HYDRUS-1D simulations. Soil moisture and porewater salinity were measured using time-domain reflectometry and suction extractors, while laboratory analyses provided electrical conductivity and pH data. The HYDRUS-1D model was calibrated and applied to simulate three groundwater depth scenarios over a ten-year period.

**Results:**

The model accurately captured water and salt transport in the vadose zone of the cotton field, with *RMSE* values below 0.05 for soil moisture and below 0.3 for soil salinity; model performance was better in deeper soil layers. Raising the groundwater level from 5.5 m to 3 m increased water storage by 45–47 cm in the upper 3 m of soil after ten years. Salt accumulation peaked near a depth of 450 cm and intensified near the surface under shallower groundwater, doubling the salinity in the top 60 cm and shifting the soil from mildly to moderately saline.

**Conclusions:**

Groundwater depth critically affects soil water and salt redistribution in coastal saline environments. Shallow groundwater tables contribute significantly to topsoil salinization, highlighting the importance of managing groundwater levels within safe thresholds to support agricultural sustainability in susceptible regions.

## 1. Introduction

Soil salinization is a global issue, and the extent of land affected by salinity has increased significantly. Statistics indicate that the worldwide area of saline alkali land is approximately 1 billion hm^2^, expanding by 1 to 1.5 million hm^2^ annually [1]. In China, coastal areas such as the Yellow River Delta and Bohai Bay plain are predominantly composed of highly saline soil, which poses a significant challenge to sustainable agricultural development [2]. Hebei Province is situated along the Bohai Sea coast and primarily features saline coastal soil. The temperate monsoon climate in this region results in uneven precipitation, a general shortage of water resources, and excessive groundwater extraction, exacerbating the degree of seawater intrusion. To desalinate the arable layer, local farmers often resort to flood irrigation using large amounts of irrigation water to wash away soil salt. Although this irrigation method effectively leaches soil salt, it leads to substantial water wastage and increases the groundwater level. Changes in groundwater levels alter the water–salt cycle in the aeration and saturation zones, affecting vegetation [3,4] and potentially causing a series of environmental issues such as secondary salinization of soil. Therefore, groundwater changes are key factors affecting soil water and salt dynamics [5]. Investigating the hydrological cycle in the vadose zone and the migration and accumulation of soil salt due to groundwater changes is crucial for the efficient use of water resources, addressing soil salinization, restoring and enhancing the ecological environment, and guiding agricultural production practices.

Many studies have focused on the impacts of groundwater changes on soil water and salt transport. Liu et al. [6,7] explored salt accumulation on the soil surface under various shallow groundwater conditions using an indoor silt loam column test. Qiao et al. [8] used a large lysimeter to control groundwater conditions and examined the effect of brackish water irrigation on water and salt transport within the soil root layer at different groundwater depths (2, 3, and 4 m). Ba et al. [9] investigated the effects of different groundwater depths on soil moisture variation, soil water balance, and winter wheat yield by manually adjusting the groundwater level. Wang et al. [10] conducted a comparative analysis of changes in field groundwater, soil water, and surface (0–30 cm) soil salinity in shallow groundwater areas (groundwater depth ≤ 2 m) under horizontal drainage conditions. Zhang et al. [11] studied the correlation between soil salinity and groundwater depth in areas with open drainage systems. Xu et al. [12] used statistical methods to analyze the impact of groundwater depth on the spatiotemporal distribution of soil water and salt in salinized irrigation areas.

Continuous monitoring of the dynamic information of soil water and salt in the field is challenging, making it difficult to describe their changes over time. Therefore, numerical simulation is an effective method for analyzing the continuous dynamic changes in water and salt in the soil vadose zone. The HYDRUS model has attracted significant attention for its ability to simulate the transport processes of soil water and salt in the field, considering the combined effects of meteorological conditions and crop growth [13,14]. Currently, many researchers have used the HYDRUS model to investigate soil water and salt transport under various conditions, including the effects of different irrigation methods or water quality on soil water and salt transport in farmland [15–19], the impact of varying shallow groundwater conditions [20–22], and the influence of water and salt regulation on crop yield [4,23–25]. However, most studies have focused on shallow groundwater depths and water and salt transport processes within the crop root zone, with experiments typically conducted during the crop growth cycle. Few studies have examined water and salt transport processes in thick vadose zones, the fate of salt, and long-term accumulation patterns under conventional irrigation practices in fields.

In this study, we conducted a field experiment in a coastal saline soil area with deep groundwater to determine water and salt accumulation in the thick vadose zone. A HYDRUS-1D model was developed to assess its applicability. Scenario simulations were used to simulate water and salt accumulation patterns in the vadose zone under three different groundwater level change conditions, providing a reference for the prevention and control of salinization and optimal management of water resources.

## 2. Materials and methods

### 2.1. Overview of test area

**Fig 1:**
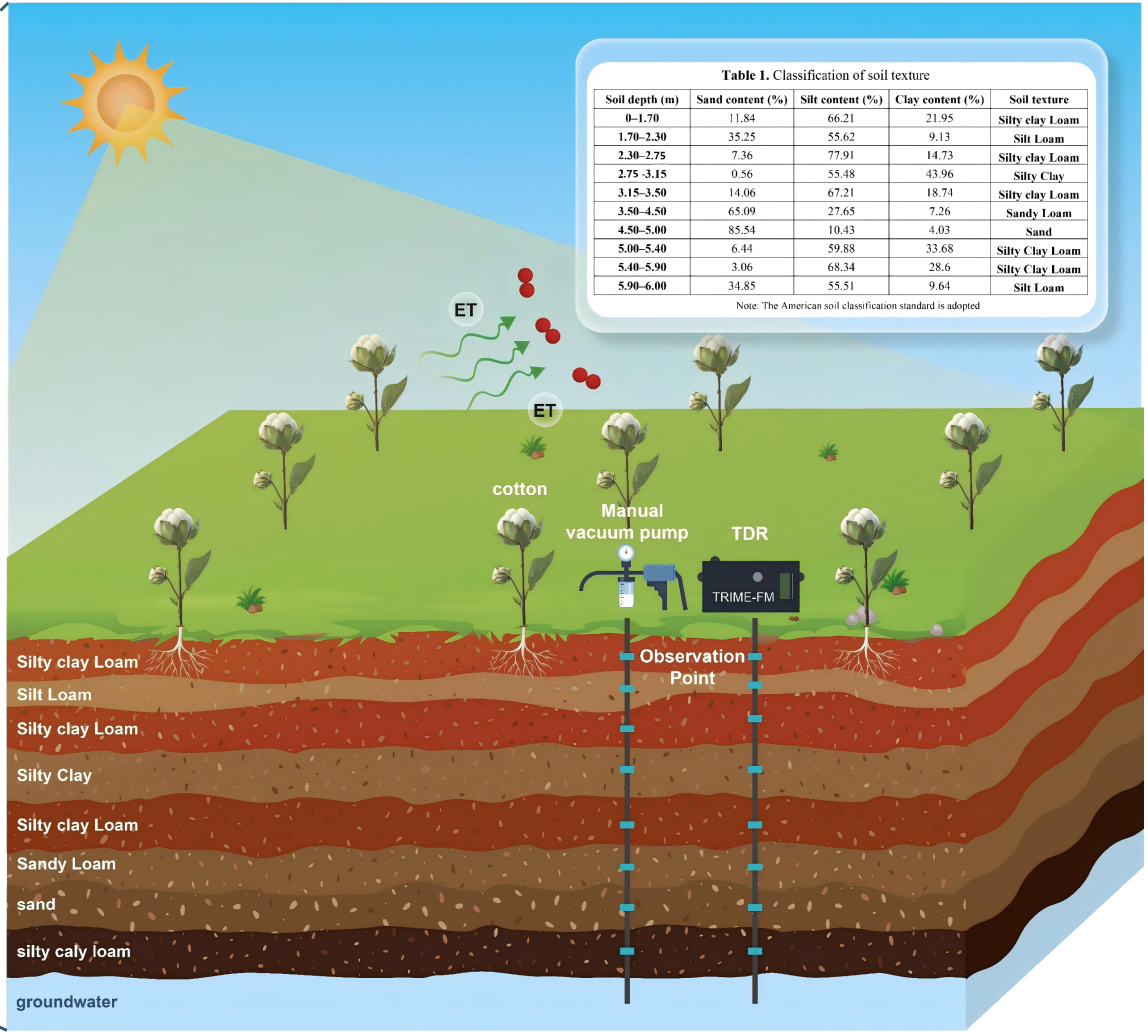
Schematic of the experimental soil profile.

The field experiment was conducted at the Nanpi Ecological Agriculture Experimental Station, Chinese Academy of Sciences, Nanpi County, Hebei Province (latitude 38°06′N, longitude 116°40′E). Schematic of the experimental soil profile in Fig 1. The experimental area is situated at an altitude of less than 20 m, featuring relatively flat terrain, and is characterised as a typical region of water scarcity and salinization near the Bohai Sea. The predominant soil types were desalted fluvoaquic soil and coastal saline soil. The cultivated soil is mostly silty clay loam, with poor soil structure and medium deviation of drainage performance. Before irrigation, the dry bulk density of the soil within a 1 m depth in the experimental area measured 1.42 g/cm^3^. The annual precipitation in this area is 400–550 mm. Influenced by the temperate monsoon climate, rainfall distribution is uneven, with the majority concentrated in summer (July and August). Historically, summer rainfall has accounted for 73% of the total annual rainfall. The remainder of the year is marked by a dry season with less rainfall, where water surface evaporation ranges from 1900 to 2200 mm, leading to significant groundwater depletion, with a current groundwater depth of 5–7 m. The area has abundant solar and thermal resources, receiving a total solar radiation of 0.267 kJ/cm^2^·h, an average of 2318 h of sunshine, and a cumulative temperature of 4300 °C for temperatures ≥10 °C.

The soil in the test area exhibited a layered structure. Before conducting the test, the layered soil in the field was analysed using a Sedima 4–12 particle size analyser (Germany). The test results are listed in Table 1. The vadose zone is divided into 10 layers, including a silty clay layer at 275–315 cm and sand layer at 350–500 cm.

**Table 1:** Classification of soil texture.

Soil depth (m)	Sand content (%)	Silt content (%)	Clay content (%)	Soil texture
**0–1.70**	11.84	66.21	21.95	**Silty Clay Loam**
**1.70–2.30**	35.25	55.62	9.13	**Silt Loam**
**2.30–2.75**	7.36	77.91	14.73	**Silty Clay Loam**
**2.75–3.15**	0.56	55.48	43.96	**Silty Clay**
**3.15–3.50**	14.06	67.21	18.74	**Silty Clay Loam**
**3.50–4.50**	65.09	27.65	7.26	**Sandy Loam**
**4.50–5.00**	85.54	10.43	4.03	**Sand**
**5.00–5.40**	6.44	59.88	33.68	**Silty Clay Loam**
**5.40–5.90**	3.06	68.34	28.6	**Silty Clay Loam**
**5.90–6.00**	34.85	55.51	9.64	**Silt Loam**

Note: The American soil classification standard is adopte

Cotton, a common cash crop in the region, was planted in the experimental area, and irrigation water was sourced from shallow groundwater extracted by a pumping well. Throughout the crop growth period, the groundwater remains at a depth of approximately 5 m year-round. Analysis of the groundwater revealed that the current total salt content was 1.0 g/L.

### 2.2. Test design

The test area spanned approximately 319.9 m^2^, with field dimensions of 16.7 m × 19.5 m. A time-domain reflectometer (TDR) and soil solution extractor were buried in a well at the centre of the field to measure soil moisture content and extract soil water, respectively. The TDR was positioned vertically and horizontally into the shaft wall at depths of 0, 20, 60, 120, 160, 200, 250, 300, 350, 400, and 450 cm. The wellhead was 1.5 m in diameter, and the length of the soil solution extractor was 1.7 m; hence, it was obliquely inserted into the soil layer, with each sampler’s insertion angle carefully controlled to maintain consistency. The burial depths of the sampling sections were 60, 120, 180, 250, 300, 350, 400, 450, and 500 cm, respectively.

Standard regional methods for irrigation and fertilisation were adopted in conjunction with the planting practices of local cotton growers. Before sowing, the soil was irrigated with 1042.5 m^3^/hm^2^ (equivalent to 69.6 m^3^/mu). Before irrigation, plant ash was sprayed evenly. After allowing the soil to dry for 4 days, it was tilled using a rotary tiller and then levelled manually, followed by artificial sowing and film mulching. Saline-alkali-resistant cotton varieties were planted in the experimental area. Before sowing, the Fengqing brand compound fertiliser was applied at a rate of 750 kg/hm^2^ (equivalent to 50 kg/mu, with 15% N, P, and K). When sowing, hole sowing was adopted, with 2–3 seeds planted under each hole, and the mulch film was 90 cm wide. Owing to the abundant precipitation throughout the cotton growing season, no additional irrigation was needed, except for the initial watering before sowing.

### 2.3. Test observation data and methods

#### 2.3.1. Meteorological data.

Meteorological observation data, including precipitation, wind speed, air temperature, ground pressure, and air humidity, were obtained from an automatic weather station in the test area. Groundwater depth and conductivity values were obtained from a dynamic water salt monitoring well.

Evapotranspiration (*ET*_*P*_), Evaporation from soil between plants (*E*_*P*_), and potential transpiration (*T*_*P*_) of crops were derived from meteorological conditions and leaf area index (*LAI*). First, the Penman–Monteith formula was applied to calculate the potential evapotranspiration (*ET*_*0*_), and then the crop *ET*_*P*_ was obtained according to the crop coefficient (*K*_*C*_). Finally, *E*_*P*_ and crop *T*_*P*_ were estimated according to the empirical formula and crop *LAI* in CERES [26].


$$ET_{p}=K_{c}\times ET_{\text{0}}.$$ (1)

$$E_{p}=ET_{p}\times \left(1-0.43LAI\right)LAI\leq \text{\textbackslash{}hspace\{0.17em}\;\}1.0$$ (2)

$$E_{p}=\frac{ET_{p}}{1.1}\times e^{-0.4\times LAI}LAI\geq \text{\textbackslash{}hspace\{0.17em}\;\}1.0$$ (3)

$$T_{p}=ET_{p}-E_{p}$$ (4)

**Fig 2:**
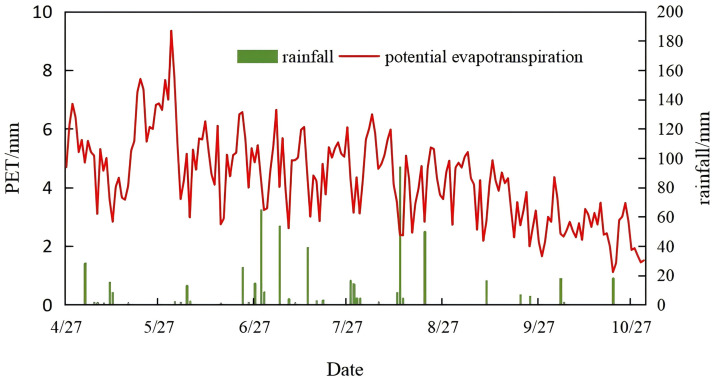
Rainfall and potential evapotranspiration during simulation.

Throughout the simulation period, the *LAI* was determined using a leaf area meter, with values ranging from 0.3 to 2.7, which was consistent with previous results [27–30]. The calculation results for crop *T*_*P*_ and rainfall during the simulation period are shown in Fig 2.

#### 2.3.2. Soil moisture and salinity.

(1) Dynamic monitoring of soil moisture

To investigate the dynamic movement behaviour of moisture in the aeration zone throughout the growth period of cotton, soil moisture was monitored using TRME-FM TDR (time-domain reflectometer) using dynamic field water and salt observation wells. The TDR probe was calibrated before formal measurement. The soil moisture content was measured once every 6 days. In the case of rainfall, the soil moisture content was measured daily and on the fourth and seventh days after rain.

(2) Collection and assay of soil solution

To monitor the dynamic movement of salt in the soil profile, soil solution samples were collected every 14 days throughout the cotton-growing period, and additional samples were collected following rainfall events. The soil solution was analysed in the laboratory to determine the EC, pH, and salinity. The EC and pH values were measured using a portable conductivity and pH meter imported from Japan (https://e.tb.cn/h.7h4aFJRUUTrlDRY?tk=4W8NUZDhdCz; Model: HORIBA PC220-k). The degree of mineralisation was determined using the gravimetric method [31].

#### 2.3.3. Crop Growth Parameters.

The crop coefficient (Kc) and leaf area index (LAI) for cotton are presented in Part A and Part B of Table 2, respectively.

**Table 2:** Crop growth and root water uptake parameters for cotton.

Part A. Crop coefficient (Kc)						
Apr	May	Jun	Jul	Aug	Sep	Oct
0.44	0.44	0.53	1.00	1.07	1.28	0.67
**Part B. Leaf area index (LAI)**						
**Jun 6**	**Jun 20**	**Jul 10**	**Jul 25**	**Aug 10**	**Aug 25**	**Sep 10**
0.30	0.64	1.60	2.70	2.10	1.60	1.60
**Part C. Feddes root water uptake parameters**						
**h_1_/cm**	**h_3_(r_2__H_)/cm**	**h_3_(r_2L_)/cm**	**h_4_/cm**	**r_2__H_/cm·d^-1^**	**r_2__L_/cm·d^-1^**	
−1	−200	−800	−8000	0.5	0.1	

Note: Data of LAI are referenced from *Source-Sink Characteristics and Yield Formation of Cotton Varieties with Different Boll Weights* (in Hebei, China); h₁, saturation point; h₃(r₂_H_), threshold at high transpiration; h₃(r₂_L_), threshold at low transpiration; h₄, wilting point; r₂_H_ and r₂_L_, maximum uptake rates at high and low transpiration demand.

### 2.4. Model construction

#### 2.4.1. Soil water movement model.

The basic equation for soil water movement is described by the Richards model by incorporating an increasing source sink term.


$$\frac{\partial \theta }{\partial t}=\frac{\partial }{\partial x}\left[k\left(\frac{\partial h}{\partial x}+cos\alpha \right)\right]-s$$ (5)

where *θ* is the soil volumetric moisture content, l; *t* is the time, d; *h* is the pressure head, cm; *x* is the spatial step size, cm; *s* is the root absorption term, cm^3^/cm^3^·d; *α* is the angle between the flow direction and the vertical direction (vertical flow α = 0; horizontal flow α = 90 °); and *k* is unsaturated hydraulic conductivity, cm/d.

#### 2.4.2 *Soil solute transport model.*

The classical convection–diffusion equation is used to describe the soil solute transport model:


$$\frac{\partial \theta c}{\partial t}=\frac{\partial }{\partial x}\left(\theta D^{w}\frac{\partial c}{\partial x}\right)-\frac{\partial qc}{\partial x}+S$$ (6)

where *C* is the solute concentration in the liquid phase (g/L), *D*^*W*^ is the hydrodynamic dispersion coefficient (cm^2^/d), *q* is the water flux obtained from the flow field, and *S* is the source–sink interaction during solute transport (complex physicochemical and biochemical processes in soil). Owing to the stability of the salt composition, the source–sink phase effect, such as adsorption during migration, was not considered.

#### 2.4.3. Root water uptake model.

In the HYDRUS model, the Feddes model is used for root water absorption function, which is based on the difference in water potential and was calculated as follows:


$$S\left(h,h_{\varphi },x\right)=\alpha \left(h,h_{\varphi },x\right)b\left(x\right)T_{p}$$ (7)

where *S* is the actual root water absorption function of crops, *α* is an empirical function related to the soil water potential, *h* is the pressure head, *b(x)* is the standard distribution function of root water uptake, *h*_*φ*_ is the seepage head related to the concentration of the soil solution, and *T*_*P*_ is crop evapotranspiration. The depth of the crop roots may vary on a daily basis as the roots develop. In the absence of field observation data, a logistic growth model [32] was used to predict root growth. It is assumed that when the growth period exceeds halfway, root growth surpasses halfway [33,34]. The root water uptake parameters for cotton are provided in Part C of Table 2.

#### 2.4.4. Initial and boundary conditions.

(1) Initial and boundary conditions of soil water movement

$$\theta (\text{x},\text{0})=\theta_{\text{i}}(\text{x})\text{ Initial conditions}$$ (8)

$$\left\{ \begin{array}{c} @lK\left(\frac{\partial h}{\partial x}+\cos\alpha \right)=q_{0}(t)\text{ second-type boundary condition}\\ h(L,0)=0\text{lower boundary condition} \end{array}\right.$$ (9)

where *X* is the spatial step size (cm), *h* is the pressure head (cm), *q*_*0*_ is the net infiltration rate (calculated as the difference between rainfall and evaporation), and *l* is the depth at which the groundwater is buried (cm). In this study, the upper boundary condition was set as the atmospheric boundary condition, which mathematically corresponds to a second-type (flux) boundary condition, with daily inputs of precipitation, potential evapotranspiration, and irrigation, while allowing for surface water accumulation; the lower boundary condition accounted for groundwater level fluctuations and was implemented as a time-varying head boundary condition.

(2) Initial and boundary conditions of soil solute transport

The initial and boundary conditions of solute transport, mainly considering the effects of precipitation, irrigation, evaporation, and groundwater level, are as follows:


$$\theta (\text{x},\text{0})=\theta_{\text{i}}(\text{x})\text{ Initial conditions}$$ (10)

$$\left\{ \begin{array}{c} @l\theta D\frac{\partial c}{\partial x}+qc=q_{0}c_{0}\text{ upper boundary condition}\\ c(x,t){|}_{x=1}=c_{d}\text{ lower boundary condition } \end{array}\right.$$ (11)

where *c* is the solute concentration of soil solution, g/cm^3^; *D* is hydrodynamic dispersion coefficient, cm^2^/d; *x* is the spatial coordinate: the origin is on the surface, and downward is positive, cm; *c*_*0*_ is the initial profile solute concentration distribution, g/cm^3^; *c*_*d*_ is the salinity of groundwater, g/cm^3^; and *l* is the buried depth of groundwater, cm.

#### 2.4.5. Model parameters.

The van Genuchten (VG) model is one of the most widely used formulas for describing the soil water retention curve, owing to its general applicability. The model is expressed as:


$$k(h)=k_{s}\frac{{\left\{\text{1}-{\left(\alpha |h|\right)}^{n-\text{1}}{\left[\text{1}+{\left(\alpha |h|\right)}^{n}\right]}^{-m}\right\}}^{\text{2}}}{{\left[\text{1}+{\left(\alpha |h|\right)}^{n}\right]}^{m\cdot l}}$$ (12)

$$k(h)=k_{s}\frac{{\left\{\text{1}-{\left(\alpha |h|\right)}^{n-\text{1}}{\left[\text{1}+{\left(\alpha |h|\right)}^{n}\right]}^{-m}\right\}}^{\text{2}}}{{\left[\text{1}+{\left(\alpha |h|\right)}^{n}\right]}^{m\cdot l}}$$ (13)

where *θ* is the volumetric water content, cm³ cm ⁻ ³; *h* is the pressure head, cm;

*θₛ,* θ*ᵣ,* α, *n, m* are parameters of the soil water retention curve, where *θₛ* is the saturated water content, *θᵣ* is the residual water content, and m = 1–1/n (with n > 1); K(h) is the unsaturated hydraulic conductivity, cmd ⁻ ¹; *Kₛ* is the saturated hydraulic conductivity, cm d ⁻ ¹; *l* is the pore-connectivity parameter.

According to the particle composition of each soil layer, the initial value of the soil hydraulic parameters was determined using the ROSETTA method in the HYDRUS-1D model, and optimised through inverse modelling [35]. The initial estimates for the five van Genuchten parameters (*θ*_*r*_, *θ*_*s*_, *α*, *n*, *K*_*s*_) were obtained using the Rosetta pedotransfer function. Inverse modelling was then performed by minimizing the discrepancy between simulated and observed soil moisture and salinity profiles. During optimization, the saturated water content (*θ*_*s*_) and residual water content (*θ*_*r*_) were primarily adjusted, while the other parameters (*α*, *n*, *K*_*s*_) were constrained within physically plausible ranges based on the Rosetta estimates. The obtained parameter values are shown in Table 3.

**Table 3:** Optimisation results of field hydraulic parameters.

Soil depth/cm	*θᵣ/*cm^3^·cm^-3^	*θₛ/*cm^3^·cm^-3^	α/cm^-1^	*n*	*K*_s_/cm·d^-1^
0–170	0.0756	0.4488	0.0055	1.6177	10.26
170–230	0.0452	0.4150	0.0052	1.6511	42.00
230–275	0.0681	0.5033	0.0059	1.6467	10.15
275–315	0.1040	0.5525	0.0125	1.4016	2.55
315–350	0.0704	0.4435	0.0050	1.6494	10.23
350–450	0.0362	0.3900	0.0316	1.4122	45.02
450–500	0.0433	0.3838	0.0394	2.0777	119.22
500–540	0.0917	0.4807	0.0087	1.4980	10.08

Note: *K*_*S*_ is the saturated hydraulic conductivity (cm·d^-1^); *θᵣ* is the residual moisture content of soil (cm^3^·cm^-3^); *θₛ* is the saturated water content (cm^3^·cm^-3^); α, n are empirical parameters

A linear relationship has been reported between the dispersion of solute transport and soil bulk density, and the initial value for solute parameters is derived using the equation d = 24.4 × unit weight – 32.34, the longitudinal dispersion [36]. The final parameters for salt migration were determined after numerous trial calculations and comparisons between the simulated and measured values. Finally, the salt migration parameters were determined and are listed in Table 4.

**Table 4:** Salt transport parameters in different soil layers.

Soil depth/cm	ρ/g·cm^-3^	D/cm	D^W^/cm^2^·d^-1^
0–170	1.40	1.80	3.2
170–230	1.37	1.80	3.2
230–275	1.50	1.60	3.2
275–315	1.60	1.10	3.2
315–350	1.50	1.70	3.2
350–450	1.33	2.36	3.2
450–500	1.20	2.50	3.2
500–540	1.50	2.00	3.2

Note: ρindicates density (g·cm^-3^); D^W^ is the molecular diffusion coefficient (cm^2^·d^-1^); D is the dispersion (cm).

#### 2.4.6. mesh discretization and time step.

The height of the model extended from the soil surface to the groundwater level. Based on the field soil texture distribution, it was divided into eight layers, and observation points were set at depths of 60, 120, 180, 250, 300, 350, 400, and 450 cm. Because of the frequent exchange of materials between the upper layer of the vadose zone and the atmosphere and active root processes, the mesh discretization follows the principle of denser units in the upper layer and sparser units in the lower layer. Specifically, 120 cm served as the dividing line, and the grid was denser above this level to account for the influence of root activity. The simulation period spanned from April 27 to October 27 (184 days). The unit of time dispersion is day, with the initial, minimum, and maximum time steps set to 0.001, 0.001, and 1 D respectively [37,38]. To improve the calculation efficiency, the HYDRUS-1D model permits automatic adjustment of the time step based on the specific conditions during the solution process.

### 2.5. Scenario setting

The verified HYDRUS-1D model was used to simulate the dynamic accumulation of water and salt in the vadose zone under varying groundwater depths, reflecting long-standing irrigation practices in the experimental area. Locally, it is customary to irrigate the soil to its moisture capacity before sowing each year and apply fertiliser. Whether additional irrigation is required during the growth period depends on the rainfall. The soil moisture was approximately 900 m^3^/hm^3^ (equivalent to 60 m^3^/mu), and the irrigation water was sourced from local groundwater, with a salinity of 1 g/L. Fertiliser was applied at a rate of 750 kg/hm^3^ (equivalent to 50 kg/mu, using a compound fertiliser containing 15% each of N, P, and K). The simulation spans 10 years using typical precipitation series years that reflect the region’s overall precipitation characteristics, and three simulation scenarios were established. (1) It was assumed that the groundwater level remained constant at 5.5 m depth over 10 years. (2) The groundwater level increased at 5.5 m to 3 m owing to inadequate drainage and other factors. (3) The groundwater level continued to decline from 5.5 m to 10 m. In the second and third scenarios, the groundwater level was assumed to fluctuate in a uniform linear manner, and each scenario was predicted and simulated separately to determine the future impact of the groundwater level trends on the water salt accumulation patterns in the vadose zone.

### 2.6. Statistical characteristics of hydrological series and selection of typical 10-year series

Rainfall records from Nanpi County, spanning 1962–2020, covering 58 years, were selected. It is assumed that this sequence of precipitation can approximately reflect the overall characteristics of precipitation in the region, and on this basis, the precipitation series was selected.

The approach for analysing representative precipitation series involves statistical parameter stability analysis and series period sliding analysis.

(1) Stability analysis of precipitation series statistical parameters

**Fig 3:**
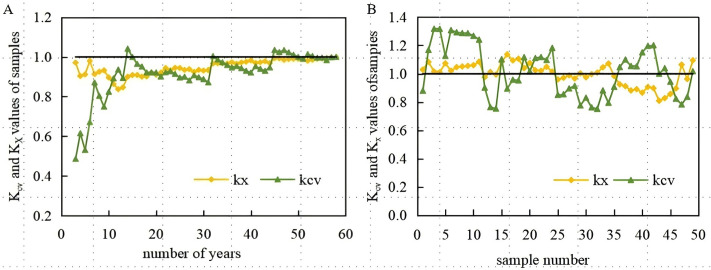
Statistical analysis of the rainfall series (stability analysis, A; sliding analysis, B).

Samples were selected from both ends of the precipitation series in the forward and reverse directions, and the number of samples increased from n = 3. The modulus coefficients *k*_*xi*_, *k*_*cvi*_, and $\overset{―}{X_{i}}$, $C_{vi}$ of the statistical parameters for each sample and the entire series were calculated as follows:


$${\overset{―}{X}}_{i}=\frac{\sum_{i=1}^{n}x_{i}}{n}$$ (14)

$$C_{vi}=\frac{\sqrt{\mathrm{\backslash raisebox}0.5em\$\sum {\left(x_{i}-{\overset{―}{X}}_{i}\right)}^{2}\$/\mathrm{\backslash raisebox}\mathrm{-0.5em}\$\left(n-1\right)\$}}{{\overset{―}{X}}_{i}}$$ (15)

$$k_{xi}=\frac{{\overset{―}{X}}_{i}}{\overset{―}{X}}$$ (16)

$$k_{cvi}=\frac{C_{vi}}{C_{v}}$$ (17)

where $\overset{―}{X_{i}}$ is the sample mean value; *X*_*i*_, sample; n, number of samples; $C_{vi}$, variation coefficient; *k*_*xi*_, mean modulus coefficient; $\overset{―}{X}$, average value of all samples; and *k*_*cvi*_, modulus ratio coefficient of variation coefficient. The average modulus coefficient *k*_*xi*_ and modulus coefficient *k*_*cvi*_,of the variation coefficient of each sample are shown in Fig 3(A).

As shown in Fig 3(A), as the number of samples increases, that is, with an increase in the number of years, the modulus coefficient gradually tends to stabilise; that is, the statistical parameters of the sample become more aligned with the statistical parameters of the population. Additionally, the larger the sample size, the closer it reflects the population characteristics.

(2) Sliding analysis of precipitation series

After establishing the duration of the simulation period, a sequence of time-sliding analyses was conducted based on this timeframe. Sliding analysis involves keeping the series length constant (the number of years of the series in this study is 10), altering the starting year sequentially, computing the statistical parameters for each sample series, and identifying the sample that most accurately represents the entire population. The results of this analysis are shown in Fig 3(B).

When selecting representative samples, it is necessary that the modulus coefficients of the mean and variation coefficients of the selected samples closely match those of the overall sample. After the calculation, the statistical parameters of the five series from 1965 to 1974, 1970–1979, 1973–1982, 1981–1990, and 1988–1997 aligned closely with the statistical parameters of the overall samples. However, the meteorological data from 1965 to 1979 were incomplete. The modulus coefficients of the mean for the 1981–1990 samples exceeded those of the 1988–1997 sample series. Therefore, after a comprehensive comparison, the 10 years from 1988 to 1997 was selected as a representative rainfall series.

### 2.7. Model performance evaluation metrics

The reliability of the model was evaluated using the mean relative error (*MRE*), root mean square error (*RMSE*), and coefficient of determination (*R²*). The specific calculation methods are as follows:


$$MRE=\frac{\text{1}}{n}\sum_{i=1}^{n}\left|\frac{y_{i}-\mathrm{\backslash stackrel}\wedge y_{i}}{y_{i}}\right|\times 100\%$$ (17)

$$RMSE=\sqrt{\frac{\text{1}}{n}\sum_{i=1}^{n}{\left(y_{i}-\mathrm{\backslash stackrel}\wedge y_{i}\right)}^{\text{2}}}$$ (18)

$$R^{\text{2}}=\text{1-}\frac{\sum_{\text{i}=\text{1}}^{\text{n}}{\left(y_{i}-\mathrm{\backslash stackrel}\wedge y_{i}\right)}^{\text{2}}}{\sum_{i=1}^{n}{\left(y_{i}-\overset{―}{y}\right)}^{\text{2}}}$$ (19)

where *y*_*i*_ is the observed value, ${\mathrm{\backslash stackrel}\wedge y}_{i}$ is the simulated value, and n is the total number of observations.$\overset{―}{y}$ is the mean of the observed values

**Fig 4:**
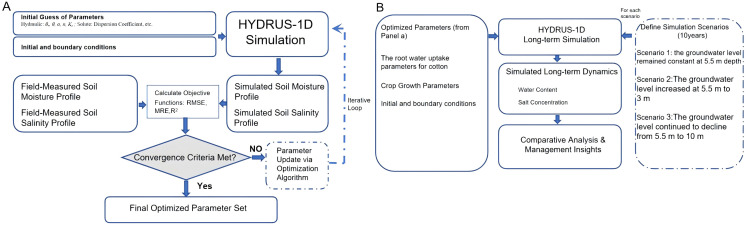
Schematic of the model calibration and scenario analysis framework. (Inverse Modeling, A; Scenario Analysis and Prediction,B).

The model configuration is illustrated in Fig 4.

### 2.8. Ethics approval and consent to participate

This study does not involve any humans or animals during experimentation, so it is not applicable in this study.

## 3. Results

### 3.1. Model reliability verification

**Fig 5:**
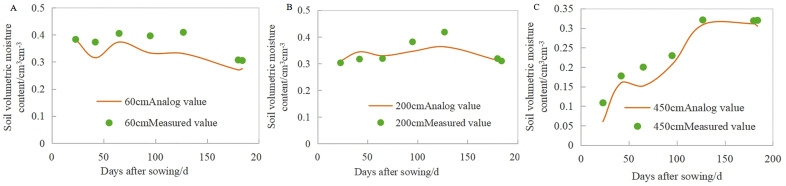
Simulated and measured values of soil moisture at different soil depths (60 cm, A; 200 cm, B; 450 cm, C).

**Fig 6:**
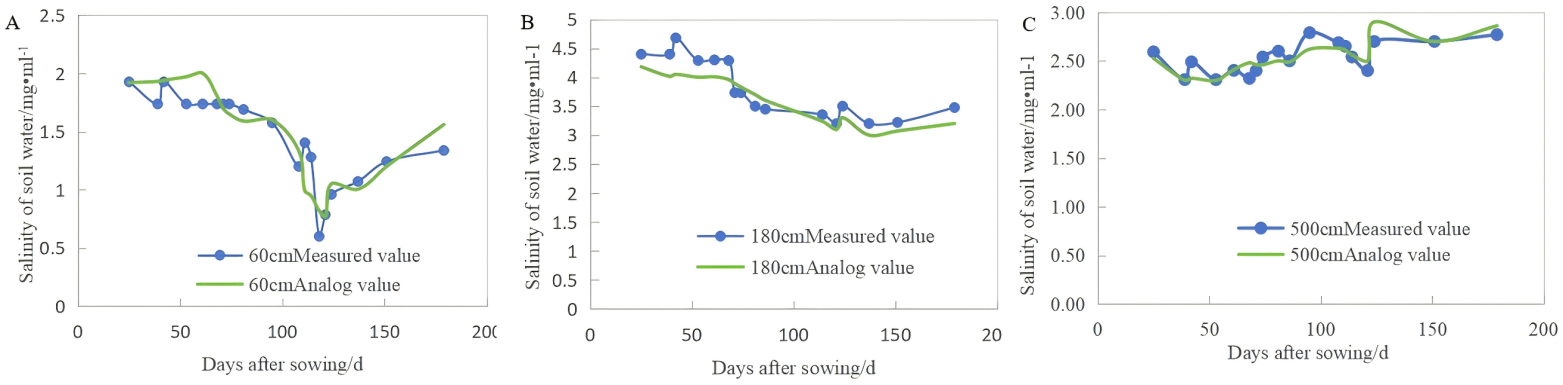
Simulated and measured values of soil water salinity at different soil depths (60 cm, A; 180 cm, B; 500 cm, C).

The validation of the model relies on measured data of soil water and salt transport in the field. HYDRUS-1D was used to simulate the moisture and salt levels in the soil layer from 0 to 500 cm during the cotton-growing period, and the results were compared with the measured values from various soil layers in the vadose zone, as shown in Figs 5,6. To assess the alignment between the simulated and observed values, metrics such as the average relative error (*MRE*), root mean square error (*RMSE*), and coefficient of determination (*R*^*2*^) were used, with the findings shown in Table 5. The *R²*, *RMSE*, and *MRE* for simulating soil moisture content at different depths of the vadose zone ranged from 0.68 to 0.99, 0.027 to 0.049 cm^3 cm-3^, and 0.020 to 0.043, respectively. In general, the simulated values of soil moisture content in different soil layers were in good agreement with the measured values. The *R*^*2*^, *RMSE*, and *MRE* between the simulated and measured values of salt content in the different soil layers were 0.66–0.82, 0.098–0.27 cm^3 cm-3^, and 0.075–0.237, respectively. Overall, the trends in the simulated and measured soil water salinity were similar, indicating that the model fits well. The *RMSE* value was less than 0.3, with even lower values for deep soil moisture and salt content, suggesting that the model performs better in deeper layers. In general, under reasonable boundary conditions and parameter selection, the simulated values of the model align with the measured values, making it suitable for simulating soil water and salt transport processes in the vadose zone of coastal saline soil areas.

**Table 5:** Statistical evaluation of model performance for soil moisture and salinity simulation.

Parameter	Simulated depth (cm)	Evaluating indicator		
		R^2^	RMSE/cm^3 cm-3^	MRE
Soil moisture content	60	0.68	0.049	0.043
	200	0.71	0.027	0.020
	450	0.99	0.029	0.024
Salt content	60	0.82	0.177	0.135
	180	0.81	0.270	0.237
	500	0.66	0.098	0.075

Note: *MRE*, the mean relative error; *RMSE,* root mean square error; *R²*, coefficient of determination

### 3.2. Prediction and analysis of water and salt accumulation in the vadose zone in 10 years under different groundwater depths

#### 3.2.1. Prediction and analysis of water movement in the vadose zone in 10 years under different groundwater depths.

**Fig 7:**
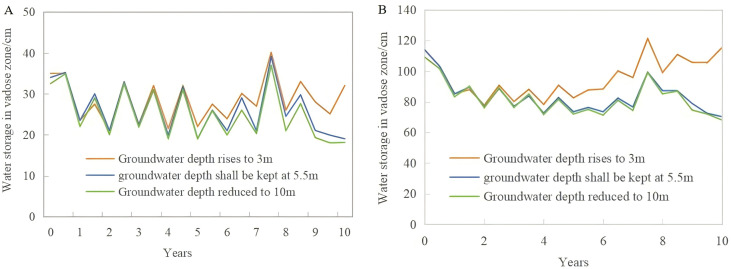
Variations in water storage in the vadose zone under different groundwater depths (1m, A; 3m, B).

The changes in water storage in the vadose zone over 10 years under the three scenarios were compared (Fig 7). To facilitate this comparison, because the lower boundary of the soil is dynamic, depths of 1 m and 3 m were chosen as the depth of the evaluation layer. Water storage variations at these depths were calculated for each of the three scenarios. Alterations in groundwater depth can significantly affect the change in water storage in the vadose zone.

By comparing Figs 7(A), (B), a consistent pattern emerges: each year, the onset of the rainy season leads to an increase in water storage in the vadose zone, whereas the conclusion of the rainy season results in a gradual decrease, indicating seasonal variations. The fluctuation in water storage in the vadose zone within 1 m is significantly affected by precipitation. Additionally, the varying groundwater conditions lead to distinct characteristics.

**Scenario 1:** When groundwater depth remained unchanged at 5.5 m, the water storage in the vadose zone generally declined with time, and there was a rapid recovery process after the 7th year. This is because the annual rainfall in the seventh year is 859 mm, which is the highest rainfall in the past 10 years, resulting in a sudden in-crease in water storage in the vadose zone.

**Scenario 2:** As the groundwater depth gradually increased from 5.5 m to 3 m, there was a noticeable annual increase in water storage in the vadose zone. By the end of the 10th year, the water storage in the vadose zone at a depth of 3 m evaluation layer reached 115.3 cm, which is 1.57 cm higher than that at the start of the first year, marking an increase of 1.4%. Additionally, with a gradual increase in groundwater burial depth, the upward recharge effect of groundwater became apparent. Under the three simulation scenarios, the water storage change curves in the vadose zone remained largely consistent with previous changes, and a clear upward trend in the water storage curve emerged as the groundwater level increased. By the end of the 10th year, the water storage in the vadose zone at the 3 m evaluation layer increased by 45.05 cm compared to that when the groundwater depth remained unchanged and by 47.3 cm compared to that when the groundwater depth decreased to 10 m.

**Scenario 3:** As the groundwater depth gradually decreased from 5.5 m to 10 m, the water storage in the vadose zone showed an overall decreasing trend. However, in the 7th year, there was a rapid increase in water storage in the vadose zone, suggesting that rainfall had a significant impact on changes in water storage in the vadose zone. Additionally, as the groundwater level decreased, the water storage within the top 3 m soil layer was slightly lower than that when the groundwater depth was maintained at 5.5 m. This indicates that when the groundwater is deeper, fluctuations in its level do not significantly affect the water storage in the top 3 m of the soil and above.

#### 3.2.2. Prediction of salt accumulation in the vadose zone in 10 years under different groundwater depths.

**Fig 8:**
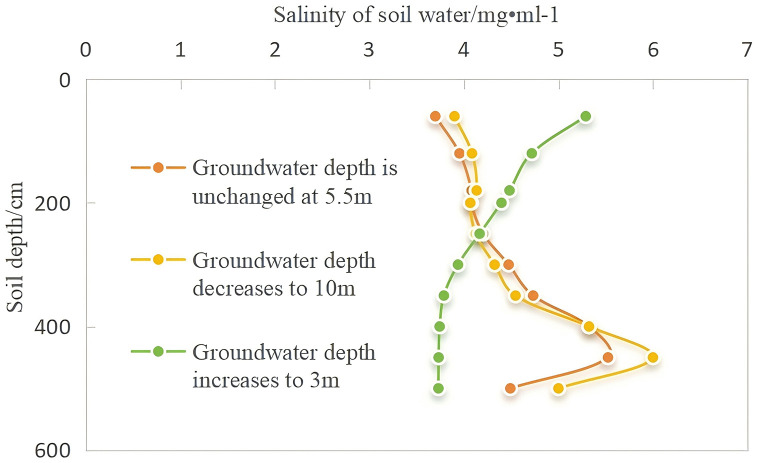
Salt accumulation patterns under different groundwater depths.

The salt accumulation in the vadose zone was simulated under three different scenarios of groundwater level changes, and the salt migration in the vadose zone at the end of the 10th year is depicted in Fig 8. The following conclusions were drawn:

**Scenario 1:** When the groundwater depth remained constant at 5.5 m, a salt con-centration peak formed in the aeration zone, with the soil water salinity reaching 5.5 g/L. After 10 years of continuous conventional irrigation, the salinity of surface soil water can reach 3.7 g/L, indicating moderate salinization.

**Scenario 2:** When the groundwater depth gradually rising from 5.5 m to 3 m, the shallow depth of the groundwater leads to frequent water exchange between the groundwater and surface. With the increase in groundwater level, the salinity of surface soil water increased significantly, and the rising trend was much greater than that in the other two scenarios. The salinity of the surface soil water reached 5.3 g/L, indicating moderate salinization. Because of the elevation in the groundwater level in the deeper layers of the vadose zone, the soil water content was high, causing a decrease in salinity concentration in the soil water, and the salt peak near the 450 cm soil layer gradually disappeared.

**Scenario 3:** When the groundwater depth continued to decrease from 5.5 m to 10 m, the salt cannot be rapidly transferred into the groundwater, resulting in soil water salinity with the salt peak reaching 6 g/L.

**Fig 9:**
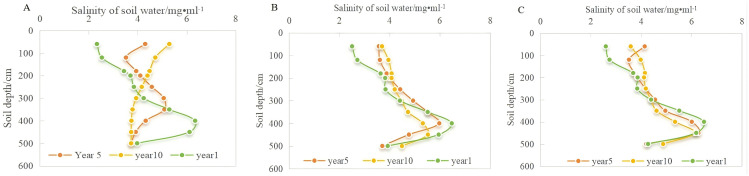
Salt accumulation trends in soil profiles over different years under varying groundwater depths. (A, rising from 5.5 m to 3 m; B, unchanged at 5.5 m; C, decreased to 10 m,).

Fig 9 illustrates the comparison of salt concentrations in the soil profiles across different years for the three groundwater depths during the simulation period. The simulation results at the conclusion of the 1st, 5th, and 10th years are shown in the figure.

**Fig 10:**
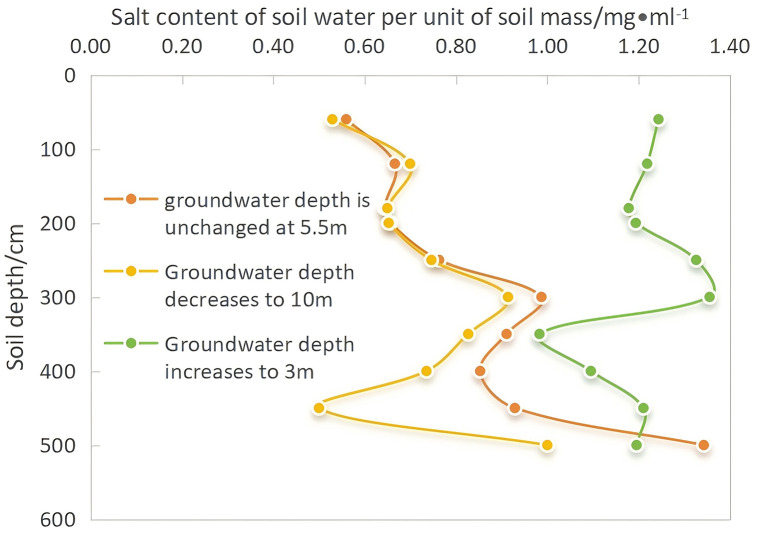
Soil profile salinity distribution after 10 years under different groundwater depths.

A common pattern is evident in Fig 10: because of evaporation and crop roots, the soil surface salt concentration increased over time compared to the value at the end of the first year under the conventional irrigation mode. Under the three simulation scenarios, the salt concentration near the 60 cm soil layer increased by 123%, 48%, and 50%, respectively, with varying groundwater depths influencing these outcomes differently.

(1) As the groundwater depth increased from 5.5 m to 3 m, the salt peak in the 450 cm soil layer gradually disappeared over time, but the rise in groundwater level also exacerbated the surface accumulation of salt. By the end of the 10th year, the surface salt concentration increased from 1.93 g/L at the beginning of the first year to 5.3 g/L.
(2) When the groundwater level remained constant at 5.5 m, the peak concentration decreased slowly over time; however, the reduction was minimal. A noticeable salt concentration peak persisted near the 450 cm soil layer in the 10th year, with substantial salt accumulation in the deeper vadose zone. Compared to that at the end of the first year, the salt concentration in the 60 cm soil layer increased slightly by 1.19 g/L, indicating that even if the groundwater level did not change significantly, strong soil evaporation in the north, coupled with unchanged irrigation methods and lack of salinization control, would still exacerbate topsoil salinization.
(3) When the groundwater depth decreased from 5.5 m to 10 m, the salt peak concentration near the 450 cm soil layer remained prominent compared to that when the groundwater level was unchanged. Salt tends to accumulate in sandy loam and sandy soils [39]. The soil texture distribution in the test area shows that the soil near 450 cm is sandy loam and sandy, indicating that soil texture is another factor influencing salt accumulation in the vadose zone. We assumed that the soil layer below 5.5 m is loamy, but the actual soil layer may be more complex. Therefore, it is likely to lead to pronounced salt accumulation in this layer.

Variations in groundwater levels affect the movement of soil water, leading to the conversion of the salt concentration in profile soil water into salt content per unit of soil water, as shown in Fig 10. This figure shows the profile of the salt distribution after 10 years under the three groundwater depth scenarios, clearly highlighting the peak of salt accumulation. In particular, when the local groundwater depth increased to 3 m, the salt peak shifted upward owing to the upward pressure from capillary rise of groundwater, resulting in a substantially higher salt content than that in the other two scenarios.

## 4. Discussion

### 4.1. Response of water and salt accumulation to groundwater level changes in coastal saline soils

The dynamics of water and salt in the vadose zone are critical in coastal saline soil regions due to their profound impact on crop productivity and long-term soil health [40,41]. Our simulation results, based on field data from Nanpi County and long-term numerical experiments using the HYDRUS-1D model, clearly demonstrate that changes in groundwater depth exert significant influence on both water storage and salt distribution within the soil profile. When the groundwater level rose from 5.5 m to 3.0 m, a substantial increase in water storage was observed within the 3 m soil layer, increasing by 45.05 cm and 47.3 cm compared to groundwater depths of 5.5 m (constant) and 10 m (declining), respectively, over a 10-year period. These results confirm that a shallower groundwater table enhances capillary rise, thereby increasing water availability in the root zone.

However, this upward flux of groundwater also brings with it dissolved salts, especially in sandy loam and sandy soils with relatively high permeability [42–44]. Notably, salt accumulation peaked near 450 cm and was particularly significant in the top-soil (0–60 cm) under the scenario of rising groundwater levels. Salinity in the topsoil doubled after ten years, indicating a shift from mild to moderate salinization. In contrast, deeper groundwater levels (>5.5 m) had limited impact on soil water and salt distribution above 3 m, highlighting the protective buffering capacity of a deeper vadose zone. This dual impact, enhanced water availability but increased salinization, underscores the delicate balance required in managing groundwater depth in coastal saline areas. While a higher groundwater table may alleviate short-term drought stress for crops, it also exacerbates long-term salt accumulation, which can negatively affect soil structure, nutrient availability, and plant health [45,46]. Thus, a dynamic and context-specific management strategy is essential, especially under climate variability and anthropogenic disturbances such as irrigation and drainage practices.

### 4.2. Applicability and performance of the HYDRUS-1D model

The HYDRUS-1D model demonstrated strong performance in simulating soil water and salt dynamics in the vadose zone under varied groundwater scenarios, which is similar to previous studies on other situations [47–49]. Model validation using measured field data across multiple soil layers (60, 200, 450 cm for moisture; 60, 180, 500 cm for salinity) indicated high simulation accuracy. The coefficient of determination (*R²*) for soil moisture ranged from 0.68 to 0.99, and *RMSE* remained below 0.05, suggesting excellent model performance, particularly in deeper soil layers. Similarly, for salt content, *R²* values ranged from 0.66 to 0.82, and *RMSE* was consistently below 0.3, meeting commonly accepted standards for environmental simulation.

Importantly, the model captured the seasonal variations in water storage associated with precipitation, as well as the cumulative effects of groundwater level changes over decadal timescales [50]. The good agreement between simulated and observed values supports the robustness of the model’s parameterization and boundary condition settings. Moreover, the use of a representative 10-year precipitation series, select-ed through statistical stability and sliding window analyses, further enhances the credibility of the long-term simulation results. Nevertheless, some discrepancies be-tween measured and simulated values were more pronounced in the upper soil layers [51]. These deviations may result from complex surface processes not fully captured by the model, such as evaporation, preferential flow, or spatial heterogeneity in soil properties. Additionally, variability in irrigation and fertiliser application practices, which were generalized in the model setup, could also contribute to simulation uncertainty.

### 4.3. Implications and limitations

This study provides valuable insights into the long-term evolution of water and salt profiles in the vadose zone under different groundwater management regimes in coastal saline soils. The integration of field measurements and numerical simulations offers a practical framework for assessing the sustainability of groundwater use in agriculture-dominated saline environments. Our findings can inform decision-makers on the potential risks of soil salinization under shallow water tables and support the design of improved drainage and irrigation strategies. Moreover, the study showcases the applicability of HYDRUS-1D as a tool for simulating complex coupled water-salt processes in heterogeneous field conditions. The approach used here—linking long-term meteorological data, site-specific soil properties, and hydrological modelling—can be applied to other similar coastal regions facing soil salinization and groundwater management challenges [52,53].

However, the study has some limitations that should be acknowledged. First, the HYDRUS-1D model assumes one-dimensional vertical flow, which may oversimplify lateral water movement and spatial heterogeneity in real field conditions [54,55]. For regions with significant horizontal hydraulic gradients or anthropogenic influences (e.g., irrigation furrows or drainage ditches), a multidimensional modelling approach (e.g., HYDRUS-2D/3D) may yield more accurate representations. Second, the model simplifies root distribution and therefore may not capture more complex two-or three-dimensional root-soil interactions or seasonal crop rotations, which can significantly influence water and salt redistribution, especially in the rhizosphere [56,57]. Including crop-soil interactions in future modelling efforts could pro-vide a more holistic understanding of agroecological processes. Lastly, while this study used typical climate years to drive the simulation, the impacts of future climate change, such as increasing temperature, changing precipitation patterns, or sea-level rise (leading to saline intrusion), were not considered. These factors are likely to alter groundwater levels and soil salinity trends, and their inclusion in future scenario analyses would enhance the relevance of the model to future land and water resource management.

## 5. Conclusions

The HYDRUS-1D model can simulate temporal changes in the distribution of water and salt in the vadose zones of cotton fields in coastal saline-soil areas. The field experiment data confirmed that the model’s simulation results were consistent with the actual measured values, demonstrating a high degree of accuracy in simulating the transport dynamics of water and salt in the soil vadose zone. Three distinct groundwater depth scenarios were established, and typical meteorological data spanning a decade were selected based on the rainfall data of Nanpi County to represent future meteorological conditions. The conventional water and fertiliser model was used to predict and simulate the dynamic variations in water and salt in the vadose zone of a cotton field under different groundwater depth conditions using the HYDRUS-1D model. Our findings suggest the following:

(1) Alterations in groundwater depth can significantly affect water storage in the vadose zone. As the groundwater depth gradually increased from 5.5 m to 3 m, the water storage in the vadose zone increased annually. By the end of the 10th year, the water storage in the 3 m evaluation layer had increased by 45.05 cm compared to that when the groundwater depth remained unchanged at 5.5 m and by 47.3 cm compared to that when the groundwater depth decreased at 10 m. Conversely, when the groundwater depth gradually decreased from 5.5 m to 10 m, with a decline in the groundwater level, the water storage in the 3 m evaluation layer was slightly lower than that when the groundwater depth remained constant at 5.5 m, indicating that fluctuations in deep groundwater (>5 m) have minimal impact on the water storage in the 3 m and higher soil layers.
(2) Variations in groundwater levels had a significant impact on the migration and accumulation of salt in the vadose zone. When the groundwater depth increased from 5.5 m to 3 m owing to upward groundwater recharge, there was a noticeable accumulation of salt. By the end of the 10th year, the salinity of soil water at the 60 cm soil layer reached 5.3 g/L, with an increase of 3.37 g/L compared to that at the beginning of the 1st year. This change led to the transition of the topsoil from mild to moderate salinization. This indicates that an increase in groundwater levels in saline soil regions can contribute significantly to soil salinization. Salt accumulation in the vadose zone near the 450 cm soil layer was evident when the groundwater depth was maintained at 5.5 m and continued to decrease to 10 m. At this depth, the soil texture is sandy loam and sandy, which facilitates salt accumulation.

## Supporting information

S1 Data The original results of Hydrus simulation. (XLSX)

## Abbreviations

TDR: Time-domain reflectometer
ET_P_: Evapotranspiration
E_P_: Evaporation from soil between plants
T_P_: Potential transpiration
